# CD137 Signaling Promotes Endothelial Apoptosis by Inhibiting Nrf2 Pathway, and Upregulating NF-*κ*B Pathway

**DOI:** 10.1155/2020/4321912

**Published:** 2020-06-06

**Authors:** Tianxin Geng, Yang Yan, Yue Zhang, Liangjie Xu, Guangyao Zang, Jin Chuan Yan

**Affiliations:** ^1^Department of Cardiology, Affiliated Hospital of Jiangsu University, Zhenjiang, Jiangsu Province 212000, China; ^2^Department of Cardiology, Ren Ji Hospital Affiliated to Shanghai Jiao Tong University School of Medicine, Shanghai 200135, China

## Abstract

**Background:**

Endothelial dysfunction and apoptosis resulting from oxidative stress can lead to the development of atherosclerosis. Our group has previously showed that CD137 signaling contributes to the progression of atherosclerosis and the vulnerability of plaques. The aim of this study is to investigate the effects of CD137 signaling in atherosclerosis on endothelial cells (ECs) apoptosis and to explore the underlying mechanisms.

**Methods:**

Serum samples were collected from 11 patients with acute myocardial infarction and 4 controls. Peritoneal injection of agonist-CD137 recombinant protein in ApoE^−/−^ mice was used to determine whether CD137 signaling can promote apoptosis in vivo, and human umbilical vein endothelial cells treated with agonist-CD137 recombinant protein, M5580 (a Nrf2 pathway agonist) and CAPE (a NF-*κ*B pathway inhibitor) were used to explore the effect of Nrf2 and NF-*κ*B pathway in CD137 signaling-induced ECs apoptosis in vitro.

**Results:**

ELISA showed that Bcl-2 in the serum of AMI patients was lower than that of the control group, while TNF-*α* and sCD137 were higher than that of the control group. Confocal microscopy and Western blot analysis showed that the nuclear translocation of Nrf2 in the agonist-CD137 group was significantly inhibited, and the expression of its downstream antioxidant enzymes was also decreased when compared with control. Immunofluorescence and Western blot results showed that the nuclear translocation of NF-*κ*B in the agonist-CD137 group was enhanced, and ELISA results showed that the secretion of proinflammatory cytokines in the agonist-CD137 group was increased. Immunofluorescence results revealed that ROS production in the agonist-CD137 group was higher than that in control, M5580 (a Nrf2 pathway agonist) and CAPE (a NF-*κ*B pathway inhibitor) groups. In vitro studies using HUVECs and in vivo studies using high-fat-fed ApoE^−/−^ mice showed that the number of apoptotic endothelial cells was the highest in the agonist-CD137 group. By contrast, both M5580 and CAPE treatments were able to reduce CD137 induced ECs apoptosis.

**Conclusions:**

Our results showed that CD137 signaling promotes ECs apoptosis through prooxidative and proinflammatory mechanisms, mediated by Nrf2 and NF-*κ*B pathways, respectively.

## 1. Introduction

Rupture of AS plaques can lead to ASCVD events such as myocardial infarction and stroke. The occurrence of AS is an extremely complicated pathophysiological process which usually started with apoptosis of endothelial cells. Apoptosis can alter the permeability of endothelial cells and the release of lipids and inflammatory cell mediators, which lead to platelet aggregation and proliferation and migration of vascular smooth muscle cells that ultimately resulting in the formation of atherosclerotic plaques [1, 2]. Endothelial cell apoptosis has been considered as one of the early events of AS. Therefore, understanding of the potential mechanisms underlying endothelial cell apoptosis will provide a new scientific basis for ASCVD prevention and treatment.

Both environmental and intrinsic factors such oxidative stress, radiation, reactive oxygen species, and inflammatory cytokines can induce ECs apoptosis and promote the development of AS lesions. Among these factors, oxidative stress is the main cause of endothelial cell injury due to reducing the production of NO and increasing the production of ROS in endothelial cells. The imbalance of oxidation-antioxidant system leads to endothelial cell dysfunction and apoptosis. Nrf2 pathway is the most important endogenous antioxidant stress pathway that protects endothelial cells from oxidative stress-induced injury. Activation of Nrf2 activates a variety of downstream proteins such as HO-1, MnSOD, and NQO1, which are involved in promoting cell oxidation resistance and enhancing the levels of intracellular antioxidant GPx [3]. Recent studies have revealed that activation of the Nrf2 pathway can reduce cardiac toxicity and improve endothelial dysfunction in the context of oxidative stress [4, 5]. Oxidative stress can abnormally activate NF-*κ*B, which is a ubiquitous transcription factor that plays a critical role in cell proliferation, differentiation, and apoptosis. Abnormal activation of the NF-*κ*B pathway can induce endothelial cell apoptosis and subsequent local inflammation. Hence, both Nrf2 and NF-*κ*B should be explored as potential targets for the prevention of atherosclerotic factor-induced cell apoptosis and inflammation.

CD137 signaling (CD137-CD137L) is a pair of inflammatory costimulatory molecules. The cross-link between CD137, a new member of the tumor necrosis factor receptor superfamily, and its ligand CD137L can trigger a two-way signal transduction that regulates the functions of various immune cells. Our previous studies have found that activation of CD137 signaling can accelerate the formation of plaques by promoting the expression of local inflammatory factors [6], phenotypic transformation of vascular smooth muscle cells [7], and the formation of plaque calcification by regulating the exocytosis of autophagosomes and inhibiting the fusion of autophagosomes and lysosomes [8]. However, the role of CD137 signaling in cell apoptosis remains largely unknown. The aim of the present study is to investigate the role of CD137 signaling in endothelial apoptosis and to explore the potential mechanisms that might be involved in the inhibition of Nrf2 pathway also the activation of NF-*κ*B pathway.

## 2. Methods and Materials

### 2.1. Reagent and Materials

Agonist-CD137 recombinant protein was purchased from Sangon Biotech (China). CD137 inhibitor was purchased from Sigma (USA). Nrf2 agonist (Dimethyl Fumarate, M5580) was purchased from AbMole (USA). NF-*κ*B inhibitor (Caffeic acid phenethyl ester, CAPE), NF-*κ*B agonist (Asatone), ROS inhibitor (Diphenyleneiodonium chloride, DPI), and ROS agonist (DMNQ) were purchased from MedChemExpress (USA). All cell culture materials were obtained from Corning (USA). OxLDL was supplied by Yiyuan Biotechnology (China). AnnexinV/Propidium Iodide (PI) assay kit was obtained from Invitrogen (USA). ROS assay kit was supplied by Beyotime Biotechnology (China). All ELISA assay kits were purchased from MultiSciences (China). TUNEL Brigthtgreen Apoptosis Detection Kit was purchased from Vazyme (China). The EpiQuik™ Nuclear Extraction Kit was purchased from Epigentek (USA). p-NF-*κ*B was purchased from Immunoway (USA), and cleaved caspase-3 was purchased from Cell Signaling Technology (USA). Other primary and secondary antibodies were purchased from Abcam (USA). All primers were designed and synthesized by Sangon Biotech (China). RNA-Quick Purification Kit was purchased from ES Science (China). Dulbecco's modified eagle's medium (DMEM), trypsin-EDTA, penicillin/streptomycin, and fetal bovine serum (FBS) were obtained from Gibco (USA).

### 2.2. Human Studies

Serum samples from acute myocardial infarction patients (*n* = 11) and medical examination health persons (*n* = 4) were obtained from the department of cardiology, Affiliated Hospital of Jiangsu University (Zhenjiang, China), from December 2017 to December 2019. Umbilical cords of healthy pregnant women were obtained from the department of obstetrics, Affiliated Hospital of Jiangsu University. This study was approved by the Ethical Committee of Affiliated Hospital of Jiangsu University and conducted in accordance with its institutional guidelines.

### 2.3. Animal Procedures

Male, 8-week-old ApoE^−/−^ mice were obtained from Vital River Laboratories (China) and received humane care in the animal laboratory center of Jiangsu University. Forty ApoE^−/−^ mice were divided into four groups as followed: control group (normal diet) (*n* = 10), HFD group (high fat diet) (*n* = 10), agonist-CD137 group (high fat diet+10 mg/kg/day agonist-CD137 recombinant protein) (*n* = 10) and M5580 group (high fat diet+10 mg/kg/day agonist-CD137 recombinant protein +5 *μ*mol/kg/day M5580) (*n* = 10). 10 mg/kg/day agonist-CD137 recombinant protein, 5 *μ*mol/kg/day M5580, and 5 *μ*mol/kg/day CAPE were administered via daily intraperitoneal injections. Mice from each group were euthanized at for 4 weeks, and the aortas were harvested for histological evaluations. The experimental scheme was approved by the Animal Care and Use Committee of the Jiangsu University.

### 2.4. Cell Culture

The umbilical cords of young healthy pregnant women after delivery were collected under aseptic conditions. The cords were digested by 1 g/L collagenase and 2.5 g/L trypsin, and the digestive solution was collected in a centrifuge tube. After the umbilical cords were rinsed with sterile PBS twice, the rinse solution was also collected in the centrifuge tube, and the supernatant was discarded after 900 r/min centrifugation for 10 min. Culture media containing 10% fetal bovine serum, 100 *μ*g/mL streptomycin, 100 U/mL penicillin, and 2 mmol/L glutamine were added to the centrifugation tube to redissolve cells, which were then transferred to culture bottle and placed in 37°C, 5% CO_2_ incubator for culture. HUVECs were cultured for three generations and then inoculated in 6-well plates at the density of 2 × 105 for subsequent experiments.

### 2.5. ELISA Assay

On the morning of the next day after admission to the hospital, 4 mL of venous blood was extracted, centrifuged at 1500 r/min for 15 min after natural coagulation to isolate serum. Both sCD137, Bcl-2, TNF-*α* in serum and IL-6, IL-1*β*, TNF-*α* in cell culture medium were detected according to the manufacturer's protocols of ELISA assay kits.

### 2.6. Flow Cytometric Assay

The treated cells were harvested and washed twice with precooled PBS and then resuspended in 100 *μ*L of 1× binding buffer. Next, 1 × 106 cells were incubated with 5 *μ*L of FITC Annexin V and 5 *μ*L of propidium iodide (PI) working solution avoiding light for 15 min at 37°C. After adding 400 *μ*L of 1× binding buffer, the cells were tested by flow cytometry on a FACS Calibur (BD Biosciences). The data were analyzed by FlowJo 7.6.

### 2.7. Confocal Microscopy Assay

The nuclear location of Nrf2 and NF-*κ*B in HUVECs was analyzed by confocal microscopy. Treated cells were fixed in 4% paraformaldehyde in PBS and permeated with 0.1% Triton X-100 followed by sealing with 5% bovine serum albumin (BSA) for 30 min. After washing three times with PBS, the cells were incubated with primary antibodies against Nrf2 (1 : 200) or NF-*κ*B (1 : 200) for overnight at 4°C. Next day, the cells were washed three times with PBS and incubated with Goat Anti-Rabbit IgG (H&L) Alexa Fluor 594 or Goat Anti-Mouse IgG (H&L) FITC secondary antibody (1 : 200) for 2 h. Nuclei were counterstained with 4′, 6-diamidino-2-phenylindole (DAPI) or propidium iodide (PI) for 10 min. Finally, HUVECs were observed under confocal microscopy.

### 2.8. ROS Measurement

Cells were implanted in six-well plates and treated. DCFH-DA was diluted with serum-free medium in accordance with 1 : 1000, and the final concentration was 10 mol/L. Remove the cell culture solution and add the diluted DCFH-DA. Incubate the 37°C cells in the incubator for 20 minutes. The cells were washed with serum-free cell culture solution three times to fully remove the DCFH-DA that did not enter the cells, and then fluorescence microscopy was used to observe the green fluorescent images. The cells also can be detected by flow cytometry. Then, fluorescence intensity was detected by fluorescence spectrophotometry with an excitation and emission wavelength of 488 and 525 nm. Image J software was used to analyze the fluorescence intensity.

### 2.9. TUNEL Staining

Aortic sections of ApoE^−/−^ mice were dewaxed and hydrated and then fixed in 4% paraformaldehyde. The sections were permeated with 0.1% Triton X-100 followed by sealing with 5% bovine serum albumin (BSA) for 30 min and then incubated with primary antibodies against CD31 (1 : 200) for overnight at 4°C. Next day, the tissues were incubated with Goat Anti-Mouse IgG (H&L) Alexa Fluor 594 secondary antibody (1 : 200) for 1 h. Subsequently, adding TUNEL detection solutions as required by the instruction. Finally, the tissues were incubated with DAPI to stain nuclei and observed under a fluorescence microscope.

### 2.10. Nuclear and Cytosolic Protein Extraction

Nuclear and cytosolic proteins were extracted from endothelial cells using the nuclear extraction kit according to the manufactures' instructions. Bradford was used to quantify protein.

### 2.11. Western Blot

The treated cells were collected and lysed with preprepared cell lysates. Total protein was extracted, and the concentration was measured. The proteins were denatured by boiling with 5 × loading buffer and *β*-mercaptoethanol. Subsequently, the protein were separated by SDS-PAGE and transferred onto PVDF membrane, which was blocked by 5% skim milk for 2 h at room temperature. PVDF membranes were incubated with primary antibody (1 : 1000 dilution) overnight at 4°C. The membranes were washed with 1 × TBST for 3 times and incubated with the corresponding secondary antibody (dilution ratio was 1 : 5000) for 2 h at room temperature. After washing three times with 1 × TBST, the luminescent agent was added to the membranes and took photos in imaging system, and Image J software was used to process the resulting images.

### 2.12. Real-Time PCR (qPCR)

Total RNA of HUVECs in each group was extracted by RNA-Quick Purification Kit, and RNA was reversely transcribed into cDNA by Thermo Fisher RT Kit. Next, cDNA was amplified with QuantiTect SYBR Green PCR Kit. Primer sequences and amplification length are shown in Table 1. PCR reaction conditions: 95°C 15 min, 95°C 15 s, 60°C 15 s, 72°C 35 s 40 cycles. The relative expression level of mRNA was calculated by comparative Ct method, that is, the relative amount was 2 − ΔΔCt.

### 2.13. Statistics

Values were expressed as means ± standard deviation (SD). All data were from at least three independent experiments and were analyzed by SPSS version 23.0. Two variables comparisons between were analyzed using the unpaired Student's *t*-test. Multiple treatment-group comparisons among multiple were assessed by one-way ANOVA followed by a post hoc LSD test, and differences were considered significant for *P* values less than 0.05.

## 3. Results

### 3.1. AMI Patients Have Higher Serum Levels of sCD137 and TNF-*α* but Lower Serum Level of Bcl-2

15 inpatients were selected in the Affiliated Hospital of Jiangsu University (Zhenjiang, China) who underwent CAG from December 2017 to December 2019. According to the CAG results, 15 patients were divided into AMI group (with acute myocardial infarction, 11 cases) and healthy controls (*n* = 4). There was no statistical difference in age, gender, weight, smoking, hypertension, blood lipid, diabetes, and other coronary heart disease risk factors between AMI group and control group (*P* > 0.05) (Table 2).

**Figure 1:**
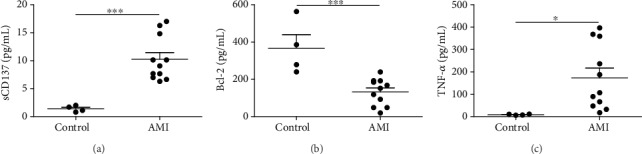
Levels of sCD137, Bcl-2, and TNF-*α* in serum from patients with acute myocardial infarction. ∗*P* < 0.05, ∗∗∗*P* < 0.001.

Bcl-2 is an inhibitor of apoptosis. Statistical analysis of ELISA showed that the serum levels of sCD137 and TNF-*α* in AMI group were higher than those in control group (Figures 1(a) and 1(c)), while Bcl-2 was lower than control group (Figure 1(b)), suggesting that CD137 may be involved in apoptosis and inflammation in AS, and could potentially affect the stability of plaques.

### 3.2. CD137 Signaling Promotes Endothelial Cells Apoptosis In Vitro

Endothelial cell apoptosis is the initial step in the cascade of events that lead to formation and rupture of AS plaque [9]. Therefore, human umbilical vein endothelial cells (HUVECs) were selected to study mechanisms of apoptosis. CD137 was little expressed in normal cells but is highly expressed when stimulated by inflammation or in AS plaques [10]. We stimulated ECs with oxLDL in vitro to simulate the high-fat microenvironment in AS plaque and to induce the apoptosis of endothelial cells.

**Figure 2:**
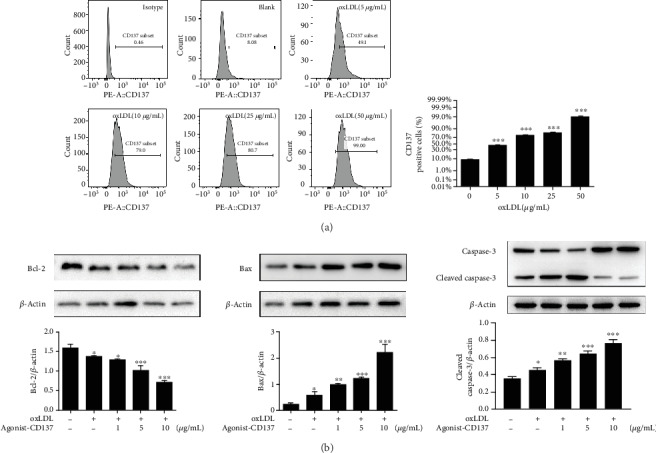
CD137 signaling induces endothelial cells apoptosis. (a) Flow cytometry for detection of CD137 expression on HUVECs. (b) Bcl-2, Bax, caspase-3, and *β*-actin protein levels by western blot analysis. The blots are representative of three independent experiments, and values are presented as the mean ± SD from three independent experiments. ∗*P* < 0.05, ∗∗*P* < 0.01, ∗∗∗*P* < 0.001 vs. control.

Flow cytometry showed that the expression of CD137 on the surface of HUVECs increased by oxLDL stimulation in a concentration—dependent manner, up to 50 *μ*g/mL (Figure 2(a)). Therefore, this concentration was selected for subsequent experiments. Similarly, Western blot revealed that treatment with CD137 agonist increased the expression of proapoptosis proteins (Cleaved Caspase-3 and Bax) and decreased apoptosis suppressor protein (Bcl-2) in HUVECs in a dose-dependent manner compared with cells stimulated by oxLDL alone (Figure 2(b)), which indicates that activation of CD137 signaling exacerbates the auxo-action of oxLDL on ECs apoptosis.

### 3.3. CD137 Signaling Inhibits Both mRNA and Protein Expression of Nrf2 Downstream Antioxidant Genes by Preventing Nuclear Translocation of Nrf2

**Figure 3:**
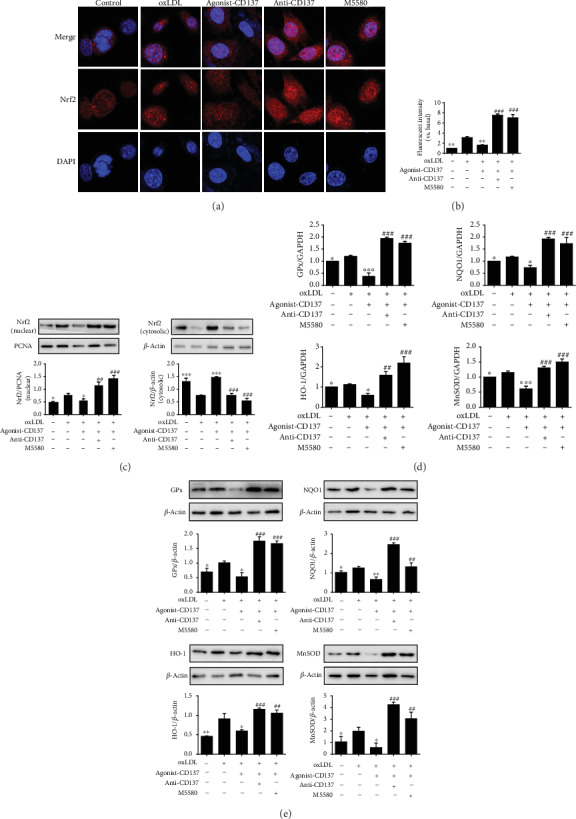
CD137 signaling inhibits Nrf2 nuclear translocation to reduce its downstream antioxidant genes and protein expression. (a) The red-stained Nrf2 moves from the cytoplasm to the nuclei, as determined by confocal microscopy. DAPI indicates 40, 6-diamidino-2-phenylindole. Magnification 630x, scale bars: 10 *μ*m. (b) Summarized data. (c) Western blot analysis tested the Nrf2 nuclear and cytosolic expression. (d) RT-qPCR evaluated the mRNA expression of the target genes (GPx, HO-1, MnSOD, and NQO1) of Nrf2. (e) Western blot evaluated the protein levels of the antioxidant enzymes (GPx, HO-1, MnSOD, and NQO1) of Nrf2. Values are presented as the mean ± SD from three independent experiments. ∗*P* < 0.05, ∗∗*P* < 0.01, ∗∗∗*P* < 0.001 vs. oxLDL-treated cells; ^#^*P* < 0.05, ^##^*P* < 0.01, ^###^*P* < 0.001 vs. agonist-CD137 group.

The nuclear factor E2-related factor 2 (Nrf2) is a key protective transcription factor against oxidative stress. Under normal conditions, most Nrf2 exists in the cytoplasm in an inactive and stable state. When stimulated by ROS, Nrf2 is translocated to the nucleus to activate the expression of downstream target antioxidant genes [3]. To verify the nuclear translocation of Nrf2, we performed immunofluorescence staining and Western blot. The staining by confocal laser scanning microscope revealed that oxLDL treatment could trigger the transfer of Nrf2 from the cytoplasm to the nucleus, while CD137 agonist pretreatment could significantly inhibit the effect. However, CD137 inhibitor or M5580 (an activator of Nrf2) pretreatment could markedly reverse the nuclear translocation of Nrf2 (Figures 3(a) and 3(b)), and the results were confirmed by Western blot (Figure 3(c)). These results indicated that CD137 signaling has inhibitory effect on the nuclear translocation of Nrf2.

As shown in Figure 3(d), pretreatment with CD137 agonist inhibited the expression of Nrf2 downstream antioxidant genes (NQO1, GPx, HO-1, and MnSOD) induced by oxLDL in HUVECs, whereas CD137 inhibitor or M5580 pretreatment significantly promoted the mRNA levels of these genes. Western blot analysis showed that CD137 signaling decreased the expression levels of NQO1, GPx, HO-1, and MnSOD, and CD137 inhibitor or M5580 increased the expression of these genes (Figure 3(e)). These data suggest that CD137 signaling can reduce the mRNA and protein expression of antioxidant genes by inhibiting Nrf2 nuclear translocation.

### 3.4. CD137 Signaling Promotes Intracellular ROS Generation and Proinflammatory Cytokines Production by Inhibiting Nrf2 Pathway

**Figure 4:**
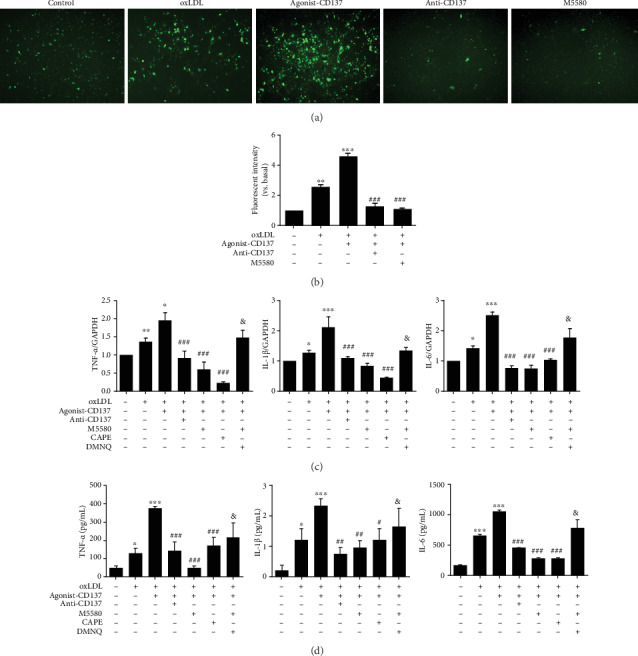
CD137 signaling promotes intracellular ROS generation and proinflammatory cytokines production by inhibiting Nrf2 pathway. (a) Analysis of ROS production by HUVECs using the DCFH-DA assay. Photographs were taken at ×100 magnification. (b) Summarized data. (c) Expression of IL-6, IL-1*β*, and TNF-*α* in HUVECs, as determined by RT-qPCR. (d) Expression of IL-6, IL-1*β*, and TNF-*α* in the supernatant of HUVECs, as determined by ELISA. Values are presented as the mean ± SD from three independent experiments. ∗*P* < 0.05, ∗∗*P* < 0.01, ∗∗∗*P* < 0.001 vs. control; ^#^*P* < 0.05, ^##^*P* < 0.01, ^###^*P* < 0.001 vs. agonist-CD137 group; ^&^*P* < 0.05 vs. M5580 group.

OxLDL binds to its receptor LOX-1 and activate NADPH oxidase to promote ROS [11]. In addition to direct damage to cells, ROS can also activate an array of oxidative stress and antioxidant stress pathways. We demonstrated that CD137 signaling can inhibit Nrf2 pathway, which is protective against oxidative stress. To prove the relationship between CD137 signaling and ROS, we conducted DCFH-DA assay to measure intracellular ROS production. Florescence intensity of ROS in CD137 agonist-stimulated HUVECs was dramatically increased compared to that of the control group (without treatment of oxLDL and CD137 agonist) and oxLDL group, but this intensity was markedly decreased by pretreatment with CD137 inhibitor or M5580 (Figures 4(a) and 4(b)). These results suggest that CD137 signaling may enhance intracellular ROS production by inhibiting the expression of antioxidant enzymes to prevent ROS clearance.

Next, we examined whether CD137 signaling could regulate production of proinflammatory cytokines by qPCR or ELISA assay. As shown in Figure 4(c), the mRNA levels of IL-6, IL-1*β*, and TNF-*α* were significantly upregulated in the CD137 agonist group compared to those in the oxLDL group. However, compared with CD137 agonist treatment, CD137 inhibitor or M5580 treatment downregulated IL-6, IL-1*β*, and TNF-*α* expression in HUVECs exposed to oxLDL-induced ROS. ELISA results further defined the role of CD137 signaling in proinflammatory cytokines secretion in HUVECs. CD137 agonist treatment significantly increased TNF-*α*, IL-6, and IL-1*β* productions in the cultured media compared to those of the oxLDL alone treatment group, and this effect was abolished by CD137 inhibitor or M5580 (Figure 4(d)).

DMNQ is a redox cycling agent that generates both superoxide and hydrogen peroxide intracellularly in a concentration dependent manner. DMNQ increases ROS generation [28]. To verify whether Nrf2 agonist M5580 reduces inflammation by inhibiting ROS, DMNQ was used to increase ROS production after M5580 treatment of endothelial cells. qPCR (Figure 4(c)) and ELISA (Figure 4(d)) showed that the levels of IL-6, IL-1*β*, and TNF-*α* in DMNQ group increased compared with the M5580 group, indicating that DMNQ could weaken the inhibitory effect of M5580 on inflammation production. These results indicate that CD137 signaling accelerates ROS generation by inhibiting Nrf2 pathway, and increased intracellular ROS may be responsible for the production of proinflammatory cytokines in endothelial cells.

### 3.5. CD137 Signaling Regulates the Secretion of Proinflammatory Cytokines by Promoting the Translocation and Phosphorylation of NF-*κ*B

**Figure 5:**
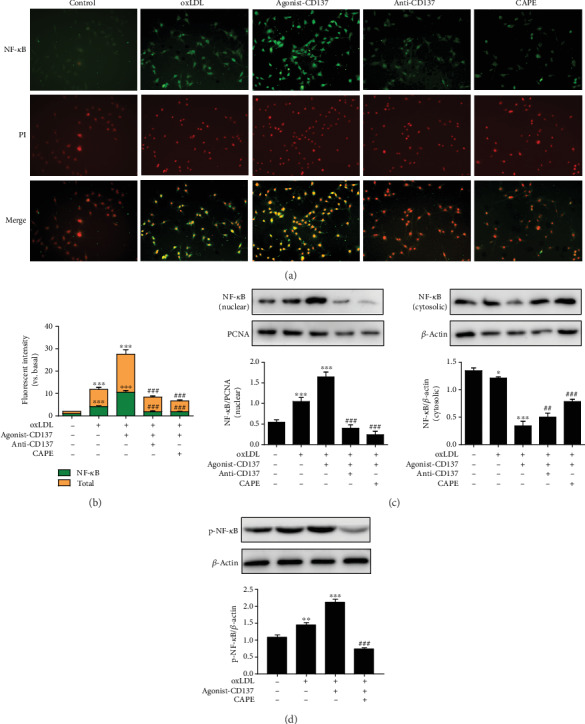
CD137 signaling promotes the translocation and phosphorylation of NF-*κ*B. (a) Images demonstrate a change in the color of the nucleus from red to yellow (due to colocalization of green FITC fluorescence and red propidium iodide fluorescence) which is indicative of NF-*κ*B translocation in HUVECs. Scale bars represent 100 *μ*m in ×200 magnifications. (b) Summarized data. (c) Western blot analysis tested the NF-*κ*B nuclear and cytosolic expression. (d) Western blot analysis tested the expression of p-NF-*κ*B. Values are presented as the mean ± SD from three independent experiments. ∗*P* < 0.05, ∗∗*P* < 0.01, ∗∗∗*P* < 0.001 vs. control; ^#^*P* < 0.05, ^##^*P* < 0.01, ^###^*P* < 0.001 vs. agonist-CD137 group.

It is generally accepted that translocation of transcription factor NF-*κ*B into the nucleus is necessary for the production of inflammatory genes [12]. To determine whether CD137 signaling could modulate NF-*κ*B translocation into the nucleus, the cells were pretreated with caffeic acid phenethyl ester (CAPE), an inhibitor of NF-*κ*B. Immunofluorescence results showed that the nucleus of agonist-CD137 group clearly changed from red to yellow, indicating NF-*κ*B translocation has occurred in these cells. However, pretreatment with inhibitor of CD137 or CAPE blocked the effect of CD137 signaling on the nuclear translocation of NF-*κ*B (Figures 5(a) and 5(b)). Moreover, this observation was supported by Western blot analysis, which revealed that CD137 signaling broadly increased the expression of NF-*κ*B in the nucleus of HUVECs but decreased upon CD137 inhibitor or CAPE treatment (Figure 5(c)). Meanwhile, Western blot result showed that the phosphorylation level of NF-*κ*B was consistent with the nuclear translocation trend (Figure 5(d)). These findings suggest that CD137 signaling could promote the activation of NF-*κ*B.

qPCR and ELISA were performed to measure the levels of proinflammatory cytokines. As shown in Figure 4(c), pretreatment with CD137 agonist increased the mRNA levels of L-6, IL-1*β*, and TNF-*α* in HUVECs in comparison with cells exposed to oxLDL alone, while pretreatment with CD137 inhibitor or CAPE decreased the mRNA levels. ELISA results showed that the expression of IL-6, IL-1*β*, and TNF-*α* in culture medium was upregulated by CD137 signaling but downregulated by CD137 inhibitor or CAPE (Figure 4(d)). These results indicated that CD137 signaling may regulate the secretion of proinflammatory factors by activating NF-*κ*B.

### 3.6. CD137 Signaling Accelerates ECs Apoptosis In Vivo and In Vitro, and the Nrf2 and NF-*κ*B Pathway Play the opposite Effect on This Process

**Figure 6:**
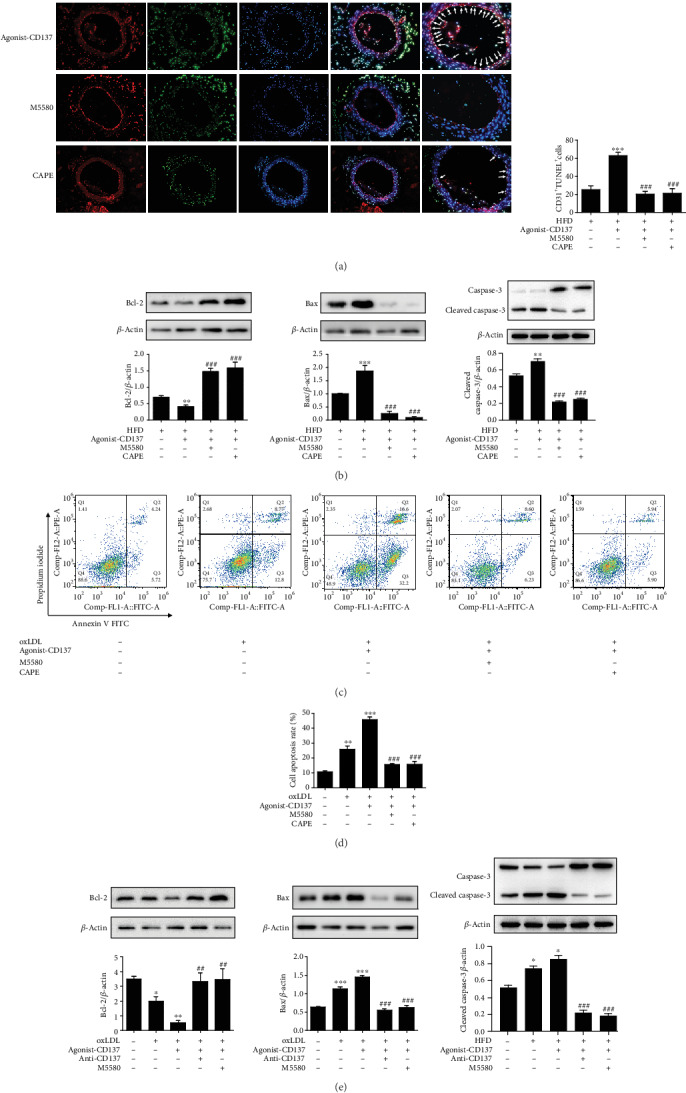
CD137 signaling accelerates endothelial cells apoptosis in vivo and in vitro, the Nrf2 and NF-*κ*B pathway play protective role in this process. (a) ApoE^−/−^ mice were fed a high-fat diet and treated with agonist-CD137 recombinant protein or/and M5580, CAPE for 4 weeks. Immunofluorescence staining of CD31 (Alexa Fluor 594 stained (red)), TUNEL stain (green), and nucleus (DAPI stained (blue)) in aortas of ApoE^−/−^ mice. Scale bars represent 50 *μ*m in ×400 magnifications. Boxed areas are shown at higher magnification in the panel aside them. Arrows indicate positivity for both CD31 and apoptotic cells. HFD indicates high-fat diet. (b) The expression of Bcl-2, Bax, and Cleaved Caspase-3 in aortas of ApoE^−/−^ mice were detected by Western blot analysis. (c) Apoptosis of HUVECs was detected by Annexin V/PI double-staining Flow cytometry. (d) Summarized data. (e) Western blot analysis determined expression of apoptosis-related proteins Bcl-2, Bax, and Cleaved Caspase-3 in HUVECs. Values are presented as the mean ± SD from three independent experiments. ∗*P* < 0.05, ∗∗*P* < 0.01, ∗∗∗*P* < 0.001 vs. control; ^#^*P* < 0.05, ^##^*P* < 0.01, ^###^*P* < 0.001 vs. agonist-CD137 group.

To elucidate the mechanism of endothelial cells apoptosis in vivo, we conducted experiments using ApoE^−/−^ mice as mentioned above. M5580 was used to activate the Nrf2 pathway. CAPE was used to inhibit the NF-*κ*B pathway. Immunofluorescence showed that high-fat diet or CD137 agonist treatment increased the number of TUNEL positive cells compared with the control group, and the agonist-CD137 group exhibited a stronger effect than the HFD group. However, the number of apoptotic cells was sharply reduced after M5580 and CAPE administration (Figure 6(a)). To further confirm the result, we analyzed the expression of apoptosis-related proteins in the aorta of ApoE^−/−^ mice in each group. As shown in Figure 6(b), Western blot showed that M5580 and CAPE reduced the activation of proapoptosis proteins (Bax and Cleaved Caspase-3) and increased the expression of antiapoptosis protein (Bcl2). These findings suggest that activation of Nrf2 pathway and inhibition of NF-*κ*B pathway can decrease the severity of CD137-induced apoptosis in atherosclerosis.

To determine the roles of Nrf2 and NF-*κ*B pathway in ECs apoptosis in vitro, we used M5580 and CAPE to offset the effect of CD137 signaling. Flow cytometry showed that CD137 signaling promoted apoptosis of HUVECs, while M5580 and CAPE dramatically reduced the percent of apoptotic cells (Figures 6(c) and 6(d)). Western blot result showed that M5580 and CAPE downregulated proapoptosis genes and upregulated antiapoptosis genes (Figure 6(e)). These results suggest that CD137 signaling promotes the apoptosis of ECs by inhibiting Nrf2 pathway and upregulating NF-*κ*B pathway.

### 3.7. Schematic Figure of Mechanism

**Figure 7:**
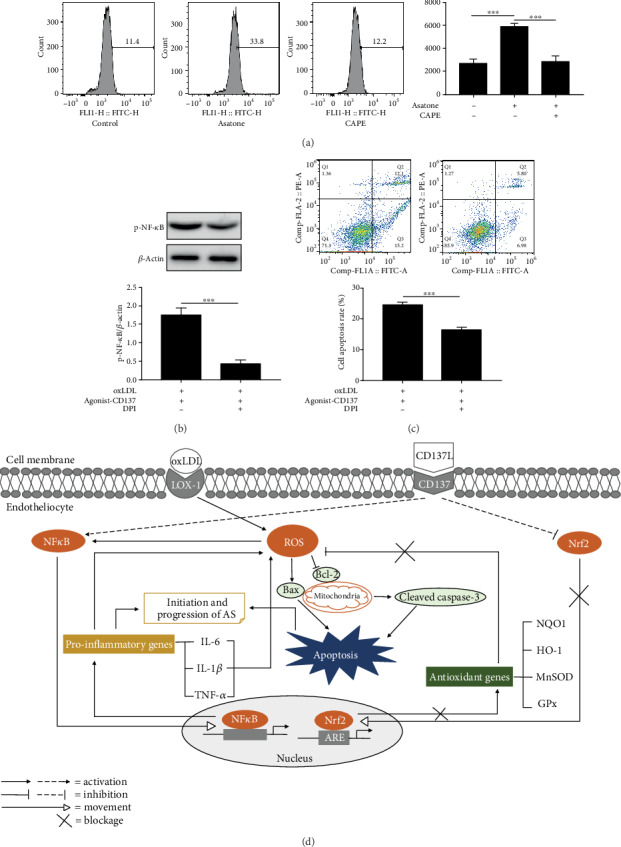
The role of ROS in endothelial cells apoptosis induced by CD137 signaling. (a) Flow cytometry evaluated ROS production by endothelial cells treated with Asatone or/and CAPE. (b) Western blot analysis tested the expression of p-NF-*κ*B. (c) Apoptosis of ECs was detected by Annexin V/PI double-staining Flow cytometry. (d) Schematic figure of mechanism. Values are presented as the mean ± SD from three independent experiments. ∗∗∗*P* < 0.001.

Figure 7(d) is a schematic diagram depicting the mechanism of CD137 signaling-mediated oxidative and inflammatory responses in ECs via Nrf2 and NF-*κ*B pathway, respectively. In agonist-CD137 condition, activated Nrf2 can translocate from nucleus to cytoplasm to increase the ROS production, and then ROS upregulates the expression of Cleaved Caspase-3 and Bax, and downregulates the expression of Bcl-2 to mediate apoptosis of endothelial cells. On the other hand, DPI (a ROS inhibitor [29]) decreasing ROS attenuated CD137 agonist-caused apoptosis (Figure 7(c)).

Moreover, the effect of NF-*κ*B pathway on CD137 agonist-induced inflammatory responses provides an attractive strategy to prevent cellular inflammation. Although it is known that NF-*κ*B promotes ROS production, it was proved by Asatone (a NF-*κ*B agonist) and CAPE (a NF-*κ*B inhibitor). Flow cytometry showed that endothelial cells in the Astone group produced significantly more ROS than the CAPE group and control group (Figure 7(a)). The effect of ROS on NF-*κ*B activation was also validated. Western blot result showed that compared with the agonist-CD137 group, the phosphorylation level of NF-*κ*B in the DPI group was significantly reduced (Figure 7(b)), indicating the interaction between ROS and NF-*κ*B.

## 4. Discussion

Apoptosis of vascular cells can increase the incidence of ACS by promoting plaque rupture, occlusive thrombosis, and embolus formation. AS is the most common disease in the cardiovascular system, and ECs injury is the initial stage of AS formation. When numerous AS-causing risk factors act on endothelial cells to cause abnormalities in their structural function and metabolism, endothelial cells lose their barrier effect and secrete a large number of biological active substances such as cytokines and inflammatory mediators [13]. Recent studies have shown that ECs apoptosis can be detected in atherosclerotic plaques, which may be an important step in the transition from stable atherosclerotic plaques to eroded plaques [14–16]. A growing number of studies have confirmed the relationship between CD137 signaling and apoptosis. One study showed that DENV induces CD137 signaling by increasing TNF-*α* production via activation of p38 MAPK to enhance hepatocyte apoptosis [30]. Another study found that activation of CD137 signaling can increase the apoptosis of vascular smooth muscle cells by decreasing the antiapoptotic regulator, Bcl-2, and upregulating cleaved caspase-3 [31]. Our previous studies have shown CD137 signaling can promote plaque formation in atherosclerosis [6], and soluble CD137 or membrane-bound CD137 expression has a positive correlation with complex coronary lesions [17, 18]. These results suggest that sCD137 may be a marker of coronary artery disease activity. Interestingly, our clinical data demonstrated that the serum level of Bcl-2 in the AMI patients was lower, while the serum levels of sCD137 and TNF-*α* were higher, compared to the control group. Therefore, we hypothesized that the process of plaque formation and even rupture mediated by CD137 signaling may be closely related to the apoptosis and inflammation of cells in blood vessels.

Our results also showed that costimulation of the agonist-CD137 recombinant protein with oxLDL reduces the expression of antiapoptotic Bcl-2 but increases the expression of proapoptotic Bax and the levels of cleaved caspase-3 in HUVECs. We further found that agonist-CD137 recombinant protein increased the number of TUNEL positive cells in atherosclerosis. To our knowledge, this is the first study to reveal the proapoptotic role of CD137 signaling in endothelial cells. In the following study, we specifically investigated the role of CD137 signaling in endothelial cell apoptosis. As expected, CD137 agonist treatment increased the number of apoptotic cells in the plaques of ApoE^−/−^ mice. This phenomenon was also verified by HUVECs cultured in vitro. Besides, CD137 signaling significantly increased the ROS production and proinflammatory factor release induced by oxLDL and decreased the expression of antioxidant genes. These alternations made by CD137 signaling could reverse by M5580, an agonist of Nrf2, or by CAPE, an inhibitor of NF-*κ*B. Therefore, the inhibition of Nrf2 and the activation of NF-*κ*B might play a critical role in the functional effect of CD137 signaling on endothelial cell apoptosis.

Various environmental and intrinsic factors in the pathological process of AS, such as mechanical stress, radiation, reactive oxygen species or nitrogen mediators, lipids, and inflammatory cytokines can mediate ECs apoptosis and promote the development of AS lesions. Among them, ROS plays an important role in vascular physiology and pathology [19]. A low dose of ROS can be used as a signal molecule in ECs to upregulate redox regulator thioredoxin and have the effect of antiapoptosis, while a high concentration of ROS can promote ECs dysfunction and even apoptosis. Activation of the CD137 signaling pathways using agonist-CD137 recombinant protein significantly induced ROS production and the levels of inflammatory mediators including IL-1*β*, IL-6, and TNF-*α* in HUVECs following oxLDL stimulation. Moreover, the expression of ROS and inflammatory cytokines was regressed by the treatment of M5580, an agonist of Nrf2, which suggested that CD137 signaling accelerates intracellular ROS production by inhibiting the antioxidant effect of Nrf2, and ROS is also the source of proinflammatory cytokines production in endothelial cells.

What is the down-stream factor that mediates CD137 induced secretion of inflammatory cytokines in HUVECs? NF-*κ*B plays an important role in the regulation of apoptosis and cell cycle. After activation, NF-*κ*B can regulate the expression of apoptosis-related proteins, such as P53, P21, Bcl-2, and Bax [20]. The most direct evidence of the antiapoptotic effect of NF-*κ*B came from RelA (p65) -deficient mice, which die due to massive apoptosis of hepatocytes in embryonic development [21]. In contrast, NF-*κ*B has been shown to promote apoptosis in some studies. Grimm reported that serum deficient medium activated NF-*κ*B and induced apoptosis of human embryonic kidney cells [22]. Huang reported that paclitaxel activates NF-*κ*B by inducing degradation of I*κ*B-*α*, thus promoting paclitaxel-induced apoptosis in solid tumor cells. The synthesis of I*κ*B-*α* induced by cortisol was found to inhibit apoptosis [23]. Thus, the role of NF-*κ*B on apoptosis may be dependent on cell types or the signal transduction pathways that were activated, or on the levels of activation or inhibition of NF-*κ*B. A recent study showed that activation of CD137 signaling could promote the expression of downstream NF-*κ*B signaling, leading to increased expression of proinflammatory cytokine IL-6 in endothelial cell-derived exosomes [37]. Tumor necrosis factor receptor-related factor (TRAF) is an adaptor protein between CD137 signaling and the NF-*κ*B pathway, acting as a “molecular switch” [38]. NF-*κ*B signaling occurs through two different pathways, both involving nuclear translocation of Rel-dimers, p50/RelA in the classical pathway, and p52/RelB in the nonclassical pathway, respectively [39]. The classical NF-*κ*B pathway mediated by TNFRs requires the recruitment of TRAF2 and cIAP proteins, which then participate in the assembly of an IKK activating complex. This complex then mediates the phosphorylation and ubiquitin-dependent degradation of the inhibitor I*κ*B and the release of NF-*κ*B. Free from its bound with I*κ*B, NF-*κ*B transported to the nucleus to activate transcription of downstream proinflammatory molecules. Activation of the nonclassical NF-*κ*B pathway relies on NF-*κ*B inducing kinase (NIK) to convert NF-*κ*B P100 into its active transcriptional regulatory fragment p52 [40]. Activation of CD137 signaling can not only initiate the classical NF-*κ*B pathway by recruiting TRAF1 or TRAF2 [41, 42] but also regulate the nonclassical NF-*κ*B pathway in T cells [43]. Interestingly, our previous study has shown that CD137 signaling can regulate NFATc1 expression in mouse VSMCs through TRAF6/NF-*κ*B p65 pathway [24], which may promote inflammation in the progression [25]. In our results, we found that CD137 signaling-dependent enhancement of nuclear translocation of NF-*κ*B in HUVECs, using CAPE, an inhibitor of NF-*κ*B, to recover the intranuclear levels of NF-*κ*B; we found that antiapoptotic gene expression was significantly reduced, while proapoptotic genes increased in both in vivo and in vitro study. We also found that the secretion of inflammatory cytokines (TNF-*α*, IL-10, IL-6) in endothelial cells was significantly reduced upon CAPE treatment.

Nrf2 is a classical anti-inflammatory and antioxidative signaling pathway and is closely implicated with the occurrence of AS. Nrf2 plays a crucial role in activating antioxidant reactions, reducing ROS production and is one of the most important transcription mechanisms to maintain the redox balance in cells [26]. Inhibition of Nrf2 signaling increases endothelial cell apoptosis [32]. Many drugs attenuate oxidative stress-induced endothelial cell injury and apoptosis by activating the Nrf2 signaling pathway and ultimately inhibit the occurrence and development of atherosclerosis [33, 34]. Recent studies have shown that activation of CD137 signaling leads to a reduction in the size and weight of lung cancer by reducing tumor proliferation and increasing oxidative stress, apoptosis, and autophagy [35]. Furthermore, one previous study showed that activation of CD137 signaling in microglia could induce oligodendrocyte apoptosis, which is mediated by ROS [36]. The antioxidant properties of Nrf2 are thought to function primarily by stimulating the transcription of antioxidant proteins. When stimulated by ROS, Nrf2 dissociates from Keap1 and becomes activated. After activation, the free Nrf2 was transferred to the nucleus and bound to the AREs to initiate the expression of downstream protective enzymes [27]. These observations correlate with our findings that CD137 signaling inhibits Nrf2 nuclear translocation in HUVECS. Furthermore, the M5580-treated HUVECs showed much stronger fluorescence intensity of Nrf2 in nucleus than the agonist-CD137 group. In addition, after applying agonist-CD137 recombinant protein treatment, the expression of Nrf2 downstream oxidative stress-related enzymes including NQO1, MnSOD, HO-1, and GPx was downregulated. Our data demonstrate that CD137 signaling may regulate the production of ROS by inhibiting the antioxidant function of Nrf2, thus promoting the apoptosis of endothelial cells. To our knowledge, this is the first study to reveal the proapoptotic role of CD137 signaling in atherosclerosis. However, our present study has a limitation that CD137 and ApoE dual gene knockout mice should be applied to validate the proapoptotic effect of CD137 signaling in vivo to fully define the role of CD137 signaling in atherosclerosis.

Notably, given that most tissues are exposed to frequently changing inflammatory and oxidative stress environments, an increasing number of studies have confirmed that there is functional crosstalk between Nrf2 and NF-*κ*B signaling pathways [44]. Activation of Nrf2 attenuates oxidative stress and neuronal apoptosis after ischemia-reperfusion injury by inhibiting NOX4/ROS/NF-*κ*B signaling [45]. In parallel, NF-*κ*B also regulates Nrf2-mediated ARE expression. A study showed that by enhancing the recruitment of histone deacetylse3 (HDAC3) in the ARE region to prevent ARE gene transcription, NF-*κ*B can inhibit NRF2 activity [46]. Crosstalk between these two pathways has become a potential target for the treatment of cerebrovascular and neurodegenerative diseases [47]. Our findings suggested that activation of CD137 signaling promotes endothelial cell apoptosis by inhibiting Nrf2 and activating NF-*κ*B signaling; however, we did not further explore whether there was an interplay between these two signalings. Additional investigations are required to find the answers to these problems.

In conclusion, we provide sufficient evidence that activation of the CD137 signal pathway can decrease the expression of oxidative stress-related enzymes, increase ROS production and inflammatory cytokines secretion, and induce endothelial cells apoptosis. The underlying mechanism is to inhibit the Nrf2 pathway and upregulate NF-*κ*B pathway. CD137 signaling might play a role in atherosclerosis via regulating the extend of endothelial cells apoptosis and inflammation, as well as the levels of intracellular ROS. These findings provide a potential method to prevent local inflammation and endothelial apoptosis in AS.

## Figures and Tables

**Table 1:** Primer sequences.

Genes	Primer sequences(5′⟶3′)
GAPDH	Forward: AACTTTGGCATTGTGGAAGG
	Reverse: ACACATTGGGGGTAGGAACA
NQO1	Forward: CCTGACATGGTCTGGGACTT
	Reverse: CAAGTTTTTGATGCCCTGGT
MnSOD	Forward: GGCCAAGGGAGATGTTACAA
	Reverse: GAACCTTGGACTCCCACAGA
HO-1	Forward: ATACCCGCTACCTGGGTGAC
	Reverse: TGTCACCCTGTGCTTGACCT
GPx	Forward: GTTTGAGAAGTGCGAAGTGAAT
	Reverse: CGGAGACCAAATGATGTACTTG
IL-6	Forward: CTCCCAACAGACCTGTCTATAC
	Reverse: CCATTGCACAACTCTTTTCTCA
IL-1*β*	Forward: AAATGCCTCGTGCTGTCTGACC
	Reverse: TCCCGACCATTGCTGTTTCCT
TNF-*α*	Forward: AAGTTCCCAAATGGGCTCCCT
	Reverse: TGAAGTGGCAAATCGGCTGAC

**Table 2:** Clinical data.

Items	AMI group (*n* = 11)	Control group (*n* = 4)
Age (years)	61.33 ± 13.16	58.00 ± 10.55
Male/female (cases)	8/4	2/2
Weight (kg)	68.00 ± 14.51	71.00 ± 8.29
Smoking [cases (%)]	7 (58.33)	2 (50.00)
Hypertension [cases (%)]	10 (83.33)	3 (75.00)
Diabetes [cases (%)]	1 (0.08)	0 (0.00)
Triglyceride (mmol/L)	1.62 ± 0.61	1.73 ± 0.81
Cholesterol (mmol/L)	4.24 ± 0.81	4.18 ± 0.87
LDL (mmol/L)	2.53 ± 0.74	2.42 ± 0.63
HDL (mmol/L)	1.09 ± 0.24	1.01 ± 0.33

## Data Availability

The data used to support the findings of this study are available from the corresponding author upon request.

## Abbreviations

AMI: Acute myocardial infarction
ASCVD: Atherosclerosis cardiovascular disease
CAG: Coronary angiogram
NO: Nitric oxide
oxLDL: Oxidized LDL (low density lipoprotein)
LOX-1: Lectinlike oxLDL receptor-1
Nrf2: Nuclear factor erythroid 2-related factor 2
ARE: Antioxidant response element
MnSOD: Manganese superoxide dismutase
GPx: Glutathione peroxidase
TUNEL: Terminal deoxynucleotidyl transferase-mediated deoxyuridine triphosphate nick-end labeling
HO-1: Heme oxygenase 1
NQO1: NAD(P)H dehydrogenase [quinone] 1
ROS: Reactive oxygen species
AS: Atherosclerosis
ECs: Endothelial cells
HDL: High-density lipoprotein
IL-6: Interleukin-6
IL-1*β*: Interleukin-1*β*
TNF-*α*: Tumor necrosis factor-*α*
CAPE: Caffeic acid phenethyl ester
sCD137: Soluble CD137
VSMC: Vascular smooth muscle cell
DPI: Diphenyleneiodonium chloride.
